# Using decision trees for measuring gender equity in the timing of angiography in patients with acute coronary syndrome: a novel approach to equity analysis

**DOI:** 10.1186/s12939-015-0280-x

**Published:** 2015-12-23

**Authors:** Arlene S. Bierman, Adalsteinn D. Brown, Carey M. Levinton

**Affiliations:** University of Toronto, 155 College Street, 3rd floor, Toronto, Ontario M5T 3M7 Canada; University of Toronto, 155 College Street 4th floor, Toronto, Ontario M5T 3M7 Canada; University of Toronto, 5 Thomas Elgie Dr. #301, Toronto, Ontario M4G 4J6 Canada

**Keywords:** Equity, Gini coefficient, Lorenz curve, Decision trees

## Abstract

**Background:**

Methods to measure or quantify equity in health care remain scarce, if not difficult to interpret. A novel method to measure health equity is presented, applied to gender health equity, and illustrated with an example of timing of angiography in patients following a hospital admission for an acute coronary syndrome.

**Methods:**

Linked administrative hospital discharge and survey data was used to identify a retrospective cohort of patients hospitalized with Acute Coronary Syndrome (ACS) between 2002 and 2008 who also responded to the Canadian Community Health Survey (CCHS), was analyzed using decision trees to determine whether gender impacted the delay to angiography following an ACS.

**Results:**

Defining a delay to angiography as 1 day or more, resulted in a non-significant difference in an equity score of 0.14 for women and 0.12 for men, where 0 and 1 represents perfect equity and inequity respectively. Using 2 and 3 day delays as a secondary outcome resulted in women and men producing scores of 0.19 and 0.17 for a 2 day delay and 0.22 and 0.23 for a 3 day delay.

**Conclusions:**

A technique developed expressly for measuring equity suggests that men and women in Ontario receive equitable care in access to angiography with respect to timeliness following an ACS.

## Background

Heart disease is the leading cause of mortality for both men and women in North American [1, 2]. Advances in clinical management coupled with increased access to timely cardiac services, such as coronary angiography, have resulted in reduced cardiac mortality. Despite these advances, it is widely recognized that inequity exists in the access that various groups have to timely cardiac services, and this may impact health outcomes [3]. In particular, previous research has shown differences in treatment patterns and health outcomes between men and women with cardiac conditions, including acute coronary syndromes [4–7], although the causes of these differences are multifactorial and may be confounded by other clinical and demographic variables [8].

Health policy researchers have developed conceptual frameworks to begin to quantify the impact of gender equity, [9, 10], created equity metrics [11–15] and refined indices used to objectively measure equity [16, 17] to answer these questions, yet none of this work has been able to determine the interactions of clinical and socio-demographic factors that may contribute to gender inequity.

To assess the impact of gender inequity on timely access to cardiac angiography for patients who suffered from an acute coronary syndrome (ACS), we used novel statistical techniques to create a general framework for measuring equity, and tested the model to ascertain whether women have inequitable access to coronary angiography compared with men. We hypothesize that once other demographic and clinical factors are controlled for, women’s access to angiography will be worse compared with men.

## Methods

All patients admitted to an acute care hospital in the province of Ontario, Canada, between the years 2002 and 2008, diagnosed with an acute coronary syndrome (ACS) and who received a coronary angiogram were eligible for entry into this study. A complete summary of the inclusion and exclusion criteria are provided in Appendix 1.

### Data sources

The Discharge Abstract Database (DAD) created, by the Canadian Institute for Health Information (CIHI) providing information on admission and discharge dates, diagnostic codes, hospital identifiers, age, sex, postal code, and discharge disposition was linked at the patient level to Statistics Canada’s Canadian Community Health Survey (CCHS) [18–21]. The CCHS survey was started in 2001 and repeated every two years. It provides information on numerous demographic metrics including language, ethnicity, cultural group, age, sex, geographic region (urban versus rural), marital status, education, residence type, labor force participation, personal and household income, and a health utility index (HUI) developed at McMaster, measuring health status. Approximately 42,000 patients were surveyed from Ontario.

### Outcome measures

The primary outcome variable, delay to procedure was defined as the difference in days between date of admission to an acute care facility and date of angiography procedure. The importance of timing of angiography results from recent studies suggesting strong correlations between early invasive treatment (i.e. angiography with revascularization if indicated) and outcomes (death, myocardial infarction, stroke, or refractory ischemia) among patients with an ACS. For some types of ACS early angiography (i.e. within 24 h of admission) is recommended to optimize outcomes [22–24], while for others a more conservative approach (i.e. three or more days) may be equally as effective [25–27]. Thus rather then use a continuous measure for delay to angiography, we chose a discrete cutpoint. Subsequently the primary outcome was defined as a binary cutoff in excess of 1 day as a delay to angiography. Secondary outcomes were defined using 2 and 3 days to angiography from admission date.

### Statistical analysis

#### Descriptive statistics

Categorical variables, both clinical and socio-economic, were compared using chi-square or Fisher’s exact tests as required. Continuous variables were reported as mean ± standard error.

### Multivariate analysis

**Fig. 1:**
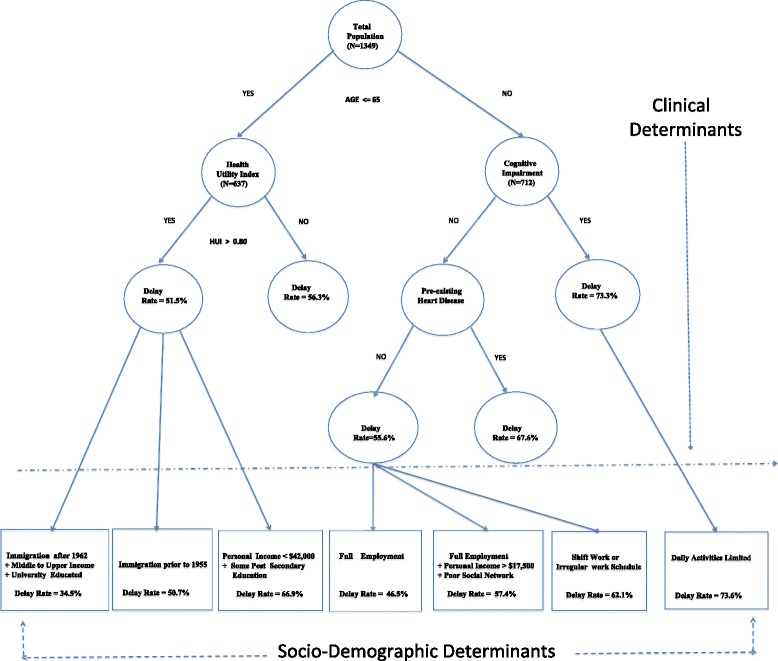
Tree construction

Decision Trees, using CART (Classification And Regression Trees) software (Salford Systems, California) [28–30] was used to examine the interactions of independent variables. The analysis begins with the complete population cohort. It then systematically chooses each available variable (continuous or categorical) and measures for each cut point its “impurity” or “disparity” in the defined distribution of the population according to some prescribed criteria, and for a given outcome measure. The criteria used to measure impurity include the Gini index. The algorithm searches amongst all the remaining “candidates” variables and selects that variable which provides the greatest degree of disparity (impurity) in the proportion of patients having a positive outcome (or negative). The form of the Gini impurity index is shown in Appendix 2 (equation 1 [29]). Once a split has occurred, the procedure is repeated in exactly the same fashion as before with the exception that the population is now defined by the population comprising the two “nodes” or subpopulations that were defined by the original split. Figure 1 illustrates the initial steps in model building. In this example with delay to angiography as an outcome (1 = No delay, 0 = delay), the most important clinical determinant of a delay occurs with a split on whether a patient age was below or above 65. Now the population is divided into two mutually exclusive subpopulations. Subsequently the next most important factor for the population aged 65 or less, is a health utility score > 0.80. Likewise the population aged above 65 has cognitive impairment as its next most important determinant with respect to delay to angiography. At this early stage of the model development we can begin to see a clear gradient of increasing delay rates going from left to right across the tree diagram. This process continues until the sample becomes sufficiently small as to render further splits unimportant. The final result is a graphical tree like structure describing a series of pathways or “branches” reflecting disparate distributions of the population with respect to the defined outcome measures. Finally, the different branches or strata are pruned back in order to further remove data artifacts and provide a more robust model (see Appendix 2, illustrating the pruning equation 2 [29]). However unlike regression modeling techniques, which simply indicate the significance levels and effect sizes, Decision Trees, or more generically recursive partition techniques, rank the overall importance of variables in their contribution towards disparities in outcomes. More practically this informs researchers and policymakers in developing strategies to target the gender equity gap, if it indeed exists.

Decision Trees allow the integration of a variety of factors reflecting clinical properties (such as co-morbid diagnoses in men and women), other social factors (such as income, education), and of the interaction of these two types of factors. In essence, each branch of the regression tree describes coefficients reflecting the rates of access formed by such factors as income or co-morbidities. The Decision Tree algorithm generates the vector of coefficients used in calculating the Gini coefficient, a cumulative measure of inequality, via a Lorenz curve [31]. If we rank order the groups corresponding to the coefficients so formed, and plot them, for example, against the cumulative rates of access to coronary angiography, the graph produced is the Lorenz curve. Furthermore, if we add a 45-degree line through the origin, representing equity, the departure of the Lorenz curve from this line characterizes the degree of inequity across groups [31]. The area between the Lorenz curve and the line of identity or equity, from here on in referred to as the departure from equity, is captured in the Gini coefficient (More precisely the Gini Coefficient is twice the difference between the Lorenz curve and the 45^0^ line of equity) whose formula can be expressed by equation 3, Appendix 2 (illustrating a more practical formulation of the Gini coefficient). The Gini coefficient can also be expressed in a more suggestive way using equation 4 [32, 33]: For purposes of inference it is much easier to work with equation 4, Appendix 2 for an alternative formulation of the Gini coefficient. The normative coefficient takes values in the range of 0 to 1, where 0 represents perfect equity and 1 represents complete inequity. By comparing the Gini coefficients (or Lorenz curves) developed around an outcome for men and women it is possible to describe the effect of gender on a health outcome. More generally, and for completeness we may generalize the Gini coefficient (Appendix 2, equation 5) by incorporating a parameter that captures the extent of aversion to gender inequitable differences [34, 35]. In the context of measuring gender equity, the algorithm is set out in the following sequence of steps:

Starting with the complete population (men and women):

1. Select a binary measure of access such as whether or not a patient had delayed access to angiography (i.e. > 1 day) following an Acute Coronary Syndrome event.
2. Construct the tree, using a Gini index as a splitting criterion, first forcing in any confounding or clinical factors including (age, type of admission, co-morbidities, health utility index (HUI), etc.).
3. Prune the tree to obtain the most parsimonious model. The strata formed by the tree yield a new set of clinical classifications [36].
4. The classifications so formed can then be ranked by increasing rates of access and compared with the cumulative rates as represented by the Lorenz curve [37]. Thus it is the contribution of decision trees in defining complex interactions or combinations of clinical factors that enhance existing approaches to evaluating gender inequities in health.
5. Run the cohort of women down the appropriate branches of the tree populating the previous defined clinical classes.
6. Repeat the same analysis for men. Consequently, Gini coefficients can be compared in order to assess the degree of disparity between men and women with respect to the particular outcome measure, adjusting or controlling for clinical variables.
7. The final stage of the model-building process is to allow all remaining variables, in this case social determinants, such as education and income levels, to enter the model. Once again pruning is performed to obtain the most robust model. The terminal nodes of the tree now define a disparate set of classes formed by the interactions of a diverse collection of variables.
8. Following a process of pruning, the cohort of men and women, separately, are run through the model emulating the same pathways as developed in the complete tree (Clinical + Social variables).
9. The distribution of individuals along matching branches can then be compared again via the Gini coefficients.

The statistical significance of differences between Gini coefficients for men and women can be determined from the standard error of the Gini coefficient itself as denoted by equation 6 [38, 39] (Appendix 2) or more generally using re-sampling techniques such as the bootstrap. Having thus defined the asymptotic standard error for the Gini coefficient, testing for differences in Gini coefficients becomes a straight forward matter of examining coverage of confidence intervals using t critical values as shown in equation 7 [38] (Appendix 2) . Furthermore, by observing the particular points on the Lorenz curve (vertical line between corresponding point for men and women) where the differences are most pronounced, we can easily isolate the particular profiles that contribute greatest towards inequity. In using t-tests we can test for statistical significance

By extension, we can incorporate into the model variables to stratify the model along regional or area axis making it possible not only to determine gender-based inequities across different outcomes but also to determine gender-based inequities across regions such as provinces. A further extension of the model allows for analysis at the group level, lending itself to embedding a hierarchical structure within the Decision Tree framework.

By the nature of the design and the way Decision Trees work, potential issues or pitfalls of confounding and effect modification have been addressed. In layering social and clinical factors, we can control for confounding. In the process of building profiles through the interactions of variables and allowing a given variable to repeatedly enter the model, effect modification is dealt with naturally.

### Ethical statement

The study was submitted through the Sunnybrook Health Sciences Centre Research Ethics Board (REB) for approval and publication. Sunnybrook Health Sciences Centre is a fully affiliated teaching hospital of the University of Toronto in Ontario, Canada.

## Results

### Study population

The study population consisted of 1349 patients of which 497 (36.8 %) were women and 852 (63.2 %) were men. Compared to men, women were older (68.3 vs. 64.3; *P* < 0.0001) had higher prevalence rates of hypertension, (57.7 % vs. 42.6 %; *P* < 0.0001), diabetes 27.2 % vs. 21.2 %; *P* = 0.01), arthritic and/or rheumatic conditions (58.2 % vs. 36.6 %; *P* < 0.0001) fair to poor self-reported health status (39.6 % vs. 34.6 %; *P* = 0.03), and lower health utility index scores [40] (0.75 vs. 0.82). Women were less likely to have a post-secondary/diploma (32.9 % vs. 44.8 %; *P* = 0.0003), married (48.3 % vs. 72.4 %; *P* < 0.0001), and have worked in the past 12 months (21.5 % vs. 47.0 %; *P* < 0.0001). Women had lower income ($20,281 vs. $38,428; *P* < 0.0001), and were more likely to report lower availability of health services (2.4 vs. 2.2; *P* = 0.02). A complete list of clinical/health and social determinants characteristics are shown in Table 1.

**Table 1:** Descriptive statistics: Clinical/Health and social determinants N (mean,standard error) for continuous predictors or N (percentage) for categorical  predictors

Variable	Description	Men	Women	*P*-Value
Clinical/Health				
Age	Age in years	852 (64.3,0.42)	497 (68.3,0.49)	<0.0001
Health utility index (Response optional)	McMaster developed health status index ranging from −0.36 (poor) to 1(perfect health)	256 (0.82,0.01)	160 (0.75,0.02)	0.01
High blood pressure	(%Yes)	363 (42.6 %)	286 (57.7 %)	<0.0001
General health	Self Reported			0.026
Poor		98 (11.5 %)	70 (14.1 %)	
Fair		197 (23.1 %)	127 (25.6 %)	
Good		301 (35.3 %)	159 (32.0 %	
Very good		193 (22.7 %)	88 (17.7 %)	
Excellent		63 (7.4 %)	53 (10.7 %)	
Previous heart disease	(%Yes)	340 (39.9 %)	213 (42.9 %)	0.302
Diabetes	(%Yes)	181 (21.2 %)	135 (27.2 %)	0.014
Arthritis/Rheumatism	(%Yes)	312 (36.6 %)	289 (58.2 %)	<0.0001
Mental health	Self Reported			0.518
Poor		13 (2.3 %)	6 (1.8 %)	
Fair		38 (6.6 %)	23 (7.0 %)	
Good		151 (26.4 %)	88 (26.9 %)	
Very good		175 (30.6 %)	115 (35.2 %)	
Excellent		195 (34.1 %)	95 (29.1 %)	
Social determinants				
Household size	Includes respondent	852 (2.2,0.04)	497 (1.80,0.04)	<0.0001
Education				
Less than secondary		278 (33.1 %)	195 (39.3 %)	0.0003
Secondary level		136 (16.2 %)	105 (21.2 %)	
Some postsecondary		58 (6.0 %)	33 (6.7 %)	
Post secondary degree		377 (44.8 %)	163 (32.9 %)	
Unmet healthcare needs		79 (9.3 %)	59 (11.9 %)	0.14
Born in Canada	(%Yes)	649 (77.6 %)	389 (78.4 %	0.78
Personal income		731 (38,428,1147)	408 (20,281,813)	<0.0001
Married	(%Yes)	616 (72.4 %)	240 (48.3 %)	<0.0001
Employed in last 12 months	(%Yes)	390 (47.0 %)	106 (21.5 %)	<0.0001
Barriers to health	(%Yes)	155 (18.9 %)	106 (21.4 %)	0.28
Rating availability of heath services	Range from 1(Excellent) to 4 (poor)	271 (2.21,0.06)	167 (2.44,0.08)	0.02
Urban residence	(%Yes)	657 (77.1 %)	402 (80.9 %)	0.11
Race: White	(%Yes)	799 (94.1 %)	476 (96.0 %)	0.16
Ethnicity				
Canadian	(%Yes)	155 (18.3 %)	104 (21.1 %)	0.22
French	(%Yes)	145 (17.2 %)	99 (20.1 %)	0.19
English	(%Yes)	283 (33.5 %)	162 (32.9 %)	0.86

### Decision tree analysis

Using Decision Tree analysis our population cohort was originally partitioned into 18 distinct patient groups (tree not shown) delineated by varying rates of delay to angiography. Due to very small numbers of events dispersed among the 18 groups, potentially resulting in spurious conclusions, the decision tree was pruned, with 7 heterogeneous groups remaining (Fig. 1). The tree shown in Fig. 1 illustrates the clinical factors (including age) forced into the tree, followed by the socio-demographic factors in the bottom portion of the graph. These groups with delay rates separated according to sex are presented in Table 2, stratified by social and clinical determinants. Going from left to right across Table 2, delays rates exhibit an increasing gradient. For example, (group 1) patient’s aged 65 or more and otherwise fairly healthy (Health Utility Index (HUI) equal to or above 0.93), middle to upper class income, immigrated after 1962 and with a post secondary education had a 34.5 % delay rate to angiography. In contrast, patients aged 65 years or higher, with cognitive impairment and/or pre-existing heart disease, and limited in daily limited activities had an overall delay rate of 73.6 % (group 7).

**Table 2:** Group profiles for Angiography delays (>1 day of index event) following an ACS event

Group # (N)	1 (171)	2 (228)	3 (75)	4 (190)	5 (116)	6 (236)	7 (333)
Clinical determinants	Age < = 65 + (HUI^*^ > = 0.93)	Age > 65 + no cognitive impairment + no previous heart disease	Age < = 65 + HUI^*^ > 0.93	Age > 65 + no cognitive impairment + no pre-existing heart disease	Age > 65 + no cognitive impairment + no pre-existing heart disease	Age < = 65 + (HUI* > = 0.80, HUI^*^ < = 0.93)	Age > 65 + cognitive impairment and/or pre-existing heart disease)
Socio-economic determinants	Immigration after 1962+ middle to upper income + University educated	Full employment	Immigration on or prior to 1955	Full employment + income > $17,500 + poor social network	Shift work or irregular schedule	Personal income < = 42,000 + some post secondary education	Daily activities limited
Delay rate							
(%)	34.5	46.5	50.7	57.4	62.1	66.9	73.6
Men (%)	36.7	48.1	52.6	58.1	63.8	70.6	71.8
Women (%)	25.0	44.4	44.4	56.1	59.6	60.2	75.7

*Health Utility Index (HUI) (−0.36 poorest health status to 1 Excellent health status)

**Fig. 2:**
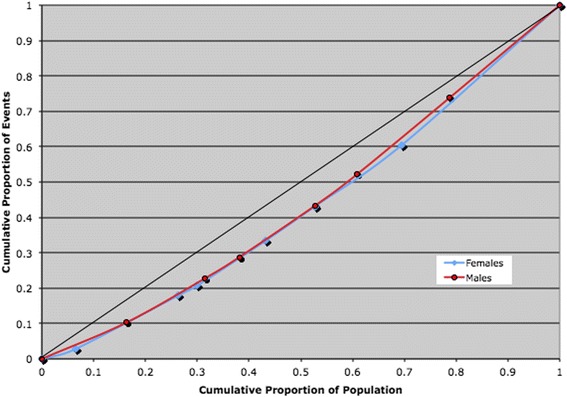
Lorenz curve comparing gender rates for Angiography with a Delay > 1 Day of an ACS (2002–2008) in Ontario (Socio-economic + health determinants)

The bottom row presents delay rates according to sex. The groups derived from the complete Decision Tree analysis tree generated the data points (delay rates for men and women) represented in Fig. 2. Figure 2 displays gender comparisons of socio-economic adjusted Lorenz curves of delays to angiography in excess of 1 day. Using the groups defined by Decision Tree analysis in the previous step, an equity measure, computed as Gini coefficients, resulted in no significant differences between men and women in delay to angiography post ACS (0.12 vs 0.14, *P* > 0.15). Geometrically, the derived value of 0.12 for men is simply the area between its Lorenz curve and the line of identity as shown in Fig. 2. Likewise, a value of 0.14 for women is similarly represented in by the Lorenz curve in Fig. 2. The difference between both curves reflect the degree of inequity between men and women. As a comparison, logistic regression models also showed gender had no significant impact on delay to angiography (OR = 0.81, *p* = 0.16) after adjusting for clinical and social determinants (Table 3).

**Table 3:** < =1 Day delay to Angiography Vs > 1 day delay to Angiography following an ACS event. Results from a logistic regression model depicting social and health determinants with Odds ratios, lower and upper confidence intervals (C.I.), and *P*-values

Variable	Odds ratio	Lower C.I.	Upper C.I.	*P*-value
Age	1.00	0.99	1.02	0.75
Sex	0.81	0.61	1.08	0.16
General health				
Poor	1.08	0.60	1.96	0.91
Fair	1.18	0.72	1.94	0.39
Good	1.09	0.69	1.71	0.84
Very good	0.97	0.61	1.55	0.48
High blood pressure	0.78	0.60	1.01	0.06
Heart disease	0.79	0.61	1.04	0.09
Diabetes	0.76	0.55	1.03	0.08
Arthritis/Rheumatism	1.00	0.77	1.30	0.98
Household Size	0.96	0.83	1.11	0.61
Education				
Less than secondary	1.45	1.07	1.96	0.11
Secondary level	1.22	0.86	1.71	0.96
Some postsecondary	1.21	0.72	2.02	0.99
Unmet healthcare needs	0.86	0.57	1.31	0.50
Born in Canada	0.59	0.42	0.83	0.00
Personal income	0.99	0.93	1.05	0.69
Married	0.93	0.69	1.27	0.66
Lifestyle improvements	1.18	0.66	2.11	0.59
Employed in last 12 months	0.82	0.57	1.17	0.26
Barriers to health	0.97	0.67	1.39	0.86
Urban residence	1.06	0.78	1.42	0.73
Race: White	2.00	1.12	3.59	0.02
Ethnicity				
Canadian	1.12	0.81	1.54	0.51
French	1.19	0.86	1.64	0.30
English	1.08	0.82	1.41	0.60

**Fig. 3:**
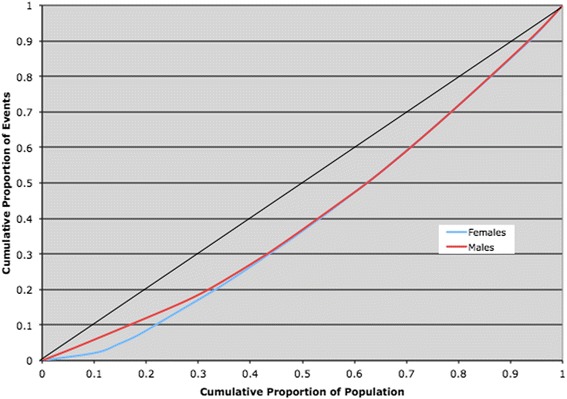
Lorenz curve comparing gender rates for Angiography with a delay > 2 days of an ACS (2002–2008) in Ontario (Socio-economic + health determinants)

**Fig. 4:**
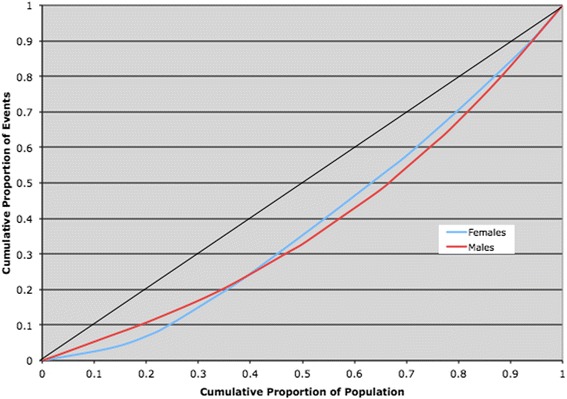
Lorenz curve comparing gender rates for Angiography with a delay > 3 days of an ACS (2002–2008) in Ontario (Socio-economic + health determinants)

When the definition of the outcome was defined as 2 or 3 days delay to angiography from hospital admission, little difference in inequity was observed. For 2 day delays the equity scores for men and women were 0.17 and 0.19 respectively (*P* > 0.10). Similarly, the difference in equity scores for a delay of 3 days was almost identical for men and women with scores of 0.23 and 0.22 respectively (*P* > 0.10). The Lorenz curves for outcomes of 2 and 3 days is shown in Figs. 3 and 4 respectively.

## Discussion

In this paper we present novel data on the impact of gender of timely access to angiography after ACS. We used a novel statistical method, along with clinical and social determinants of health to create a model to isolate the impact of gender on access to cardiac procedures, represented by coronary angiography. Our results demonstrate that when multiple clinical and socio-economic factors are controlled for, the impact of gender on time to angiography is non-significant.

The results presented in this paper using the Decision Tree analysis technique were similar to that derived from logistic regression. Why use this Decision Tree analytic technique over more traditional methods like logistic regression? Decision Trees, unlike logistic regression, present data in a visual format. For health policy makers, decision trees more readily allow greater transparency in the interpretation of the factors that results in greater or lesser health inequity. How? Simply descending the various branches of a tree reveals the interaction of socio-demographic factors that most contribute to health inequity. This is further amplified by graphically representing the results of the decision tree on a Lorenz curve stratified according to gender. Hypothetically, unlike our particular study, if a large difference occurred at a spilt represented by say, education attainment, it could be transparently depicted on the Lorenz curve. In such an instance a policy maker could focus on specific interventions for patients with low levels of educational attainment to narrow such inequities. Thus policy makers or planners could more accurately target specific subpopulations disadvantaged in their healthcare treatment, through illuminating how these clinical and social factors interact with one another.

Although, in our study, and more generally decision trees are pruned to avoid spurious findings or over fitting resulting from small sample sizes in the descending branches of the tree, they are not adversely affected by outliers as they depend on the relative values of a variable unlike traditional analytical methods including logistic regression. Moreover, the decision tree technique affirms its robustness or stability in examining the results when outcomes varied according to delay times (i.e. 1,2 or 3 days). Small changes in delay times were reflected in similar effects on the equity index scores and differences between men and women.

Unlike Logistic Regression, this technique was developed from the ground up specifically to address the measurement definition of equity as elucidated by Braveman [41] and embraced by the World Health Organization (WHO):

*“Equity is the absence of avoidable or remediable differences among groups of people, whether those groups are defined socially, economically, demographically, or geographically. Health inequities therefore involve more than inequality with respect to health determinants…….”*

The results of this study are consistent with recent studies that have demonstrated that age and pre-existing co-morbidities were independent predictors of coronary angiography following an ACS [42–44]. Although age, health, comorbidities such as cognitive impairment, and socio-economic determinants are important factors in inequity of timing to angiography, care should be taken in making any inferences as procedure appropriateness may highly influence these and many other inequities [45–47]. Nonetheless additional examinations may be warranted in order to investigate inequities resulting from these socio-economic determinants and develop interventions to reduce them. Furthermore, previous research has shown gender differences in outcomes of patients hospitalized with ACS, with both biological and sociological explanations for such differences. A landmark paper by Ayanian and colleagues [44] found women hospitalized for coronary disease in the states of Massachusetts or Maryland underwent fewer cardiac procedures than men, and more recent studies involving Medicare patients hospitalized in acute care centers across the United States have shown this same care gap, though smaller than previously reported [48]. Within Canadian jurisdictions the picture was mixed. A study by Fransoo et al. [49] analyzed a cohort of patients from the province of Manitoba, and found no gender difference in angiography rates during index hospitalization after adjusting for age. More recently, research on patients hospitalized in Alberta found significant gender differences in the timing of angiography (i.e. Using a 48 h time window from admission) adjusting for income quintiles [50]. In previous studies, women have tended to be older with more co-morbidities, which may have dissuaded clinicians from treating them as aggressively. Furthermore, social factors, such as income and education level, may confound any analysis of the impact of gender. It has also been observed that because women may manifest disease somewhat differently than men more aggressive treatment options are sometimes overlooked [51, 52]. However, once a decision is made to have patients undergo an angiogram, timing no longer differentiates men and women regardless of clinical and socio-demographic determinants, as supported by our study. Moreover once a decision is made to perform an angiography, perhaps pre-existing guidelines supersede any other factors including gender, race, education etc.

The Decision Tree modeling technique can identify and delineate cases in which biological factors have a legitimate impact on access, from cases in which social determinants (or the interaction social factors with clinical factors), have a potentially reducible impact on health inequities. This makes this statistical modeling technique ideally suited to address the complex interplay of factors that impact access to cardiac services and care. To our knowledge, this is the first study to use the Decision Tree model to assess the impact of gender inequity on access to timely angiography, and more generally in the measurement of equity itself.

## Conclusion

The statistical methods and results of this paper have significant health policy implications. The Decision Tree technique provides guidance on the specific factors within populations that programs can target to reduce inequity and can better tease out the impact of biological factors from socio-economic ones. From a policy perspective, targeting specific populations subgroups that are underserved in their health care needs is likely to be a more cost-effective approach to spending health care dollars, and will have a greater impact on health outcomes [53, 54].

This study has significant limitations that warrant mention. The interval of time between hospitalization and response to the CCHS survey may have been as much as 1 year. However many of the socio-economic characteristics including age, co-morbidities, gender, race, ethnicity, income, labor participation are fixed, or very unlikely to have changed very much within the time window of hospitalization and survey response. For the same reasons the inequities in age and socio-economic determinants are not artifacts of these time gaps. Likewise, the administrative data component of our database provided details of the diagnosis and subsequent procedures, but little information on the extent, severity of disease (i.e. single vs. multi vessel involvement), or classification of the ACS (ST-segment elevation myocardial infarction (STEMI) and Non-STEMI). Care must also be taken in generalizing these results beyond the province of Ontario. As alluded to earlier, while the results in Manitoba agreed with those of Ontario, the Alberta study diverged in this respect. Finally, data on the admitting institution – such as the presence of an angiography laboratory was not available although we were able to access data on the procedures performed in all hospitals treating the patient during the same episode of care. With respect to the analytic method itself, a potential limitation is the requirement of significant sample sizes in order to accommodate large numbers of factors and their interactions. Yet with the growing availability of big data and linked data from multiple sources including EHR’s (electronic health records), this approach and inclusion of multiple factors will become more feasible. Despite these limitations, however, the Decision Tree modeling technique and the use of the Gini coefficient provides a novel technique to identify and delineate cases in which biological factors (e.g. co-morbidities and age) have a legitimate impact on differences on measures of access, from cases in which social determinants (or the interaction of social factors with clinical factors), have a potentially reducible impact on health inequities. 

In conclusion, use of a novel statistical modeling technique has shown that timing in access to coronary angiography was not adversely affected by gender, after controlling for multiple biologic and socio-economic factors. Further research into the relationships between these factors is warranted.

## Appendix 1

**Table 4:** Inclusion, exclusion, and outcome definitions using ICD-10 codes

Numerator		
	Criteria	Codes(ICD-10)
Include:	Cases within denominator with:	
	Coronary angiography within 1 day	3.IP.10.^^
Denominator		
	Criteria	Codes
Include (from within the linked database –Discharge Abstract Data (DAD) linked to Canadian Community Health Survey):	Acute Myocardial Infarction (AMI)	I21.^, I22.^ (Diagnosis Type M (but not M and 2), Type 1 (with another Dx type M and 2))
OR	Unstable angina	I20.0
OR	Cardiogenic shock	R57.0
AND	Coronary angiography	3.IP.10.^^
Exclude:		
	Chronic renal failure/hepatic failure	K72.1, N18.^ (any diagnosis type on the abstract)
	Psychiatric disorder (excluding depressive disorder and recurrent depressive disorder)	F04-07, F09-25, F28-31, F34, F48, F50-F59, F60-F69, F70-F79, F80-F89, F90-F98

## Appendix 2

**GINI INDEX**

1 $$ \mathrm{Gini}\kern0.3em \mathrm{Index}:\mathrm{i}\left(\mathrm{t}\right)={\displaystyle \sum_{\mathrm{i}\ne \mathrm{j}}\mathrm{p}\left(\mathrm{j}\Big|\mathrm{t}\right)\mathrm{p}\left(\mathrm{i}\Big|\mathrm{t}\right)} $$

Where

t= the node being split

i,j= are those classes corresponding to the outcome measure

p(.) = probability a case is in class i, i = 1.. n

**PRUNING EQUATION**

2 $$ \mathrm{R}\left(\mathrm{t}\right)+\upalpha \left|\mathrm{T}\right| $$

Where R(t) = Σ r(t)p(t)

^t ε T^

r(t) = within node Gini diversity index

p(t) = probability of being in node t

α = complexity cost parameter

|T| = Number of terminal nodes in subtree T

**EQUIVALENT FORMULATIONS OF GINI Coefficient**

3 $$ \mathrm{G}\kern0.5em =\kern0.5em 1+1/\mathrm{N}-2/\left({\mathrm{N}}^2\upmu \right)\left\{{\mathrm{y}}_1+2*{\mathrm{y}}_2+3*{\mathrm{y}}_3+4*{\mathrm{y}}_4+5*{\mathrm{y}}_5\dots \dots \mathrm{N}*{\mathrm{y}}_{\mathrm{N}}\right\} $$

For y_1_ ≥ y_2_ ≥ y_3_ ≥ y_4_ ≥ y_5_ … … ≥ y_N_

Where y_i_ i = 1… N represent the outcame rates for each group defined by the decision tree algorithm,

μ = mean of outcome rates across all groups = $\left(1/\mathrm{N}\right)\ast {\displaystyle \sum_{\mathrm{i}}{\mathrm{y}}_{\mathrm{i}}}$

and,

N = the number of groups classified by the decision tree.

Alternatively the GINI coeffiecient can be expressed in a more suggestive way as:

4 $$ \mathrm{G}=\kern0.5em 2\ast \mathrm{Covariance}\kern0.5em \left({\mathrm{y}}_{\mathrm{i}},\mathrm{F}\left({\mathrm{y}}_{\mathrm{i}}\right)\right)/\upmu $$

Where F() is the cumulative distribution of outcome rates.

**GENERALIZED GINI Coefficient (G(*****v*****))**

$$ G(v)=-v\operatorname{cov}\left(y,{\left[1-F(y)\right]}^{v-1}\right)/\overline{y} $$

Where *v* is the inequity aversion parameter, F (.) is cumulative distribution of outcome rates, y is the average outcome rate, the covariance (cov) is:

$$ \operatorname{cov}\left(y,{\left[1-F(y)\right]}^{v-1}\right)={\displaystyle \sum_{i=1}^n{w}_i\left({y}_i-\overline{y}\right)}\left[{\left(1-{\widehat{F}}_i\right)}^{v-1}-m\right] $$

And w_i_ are individual or grouped weights with m defined as:

5 $$ m={\displaystyle \sum {w}_i{\left(1-{\widehat{F}}_i\right)}^{v-1}} $$

**STANDARD ERROR OF GINI Coefficient**

6 $$ \mathrm{s}.\mathrm{e}.\kern0.2em \left(\mathrm{Gini}\kern0.3em \mathrm{coefficient}\right)=2\ast \left(\mathrm{s}.\mathrm{e}\left(\widehat{\uptheta}\right)\right)/\mathrm{n} $$

Where:

n = Number of classes defined by tree

$\widehat{\uptheta}$ = G (Gini coefficient) or G(*v*) (Generalized Gini coefficient)

$\mathrm{s}.\mathrm{e}.\left(\widehat{\uptheta}\right)$ = standard error of ordinary weighted least squares regression predictions for Gini coefficient

**CONFIDENCE INTERVAL FOR GINI COEFFICIENTS**

7 $$ \left(\mathrm{G}\kern0.5em \hbox{-} \kern0.5em {\mathrm{t}}_{\upalpha /2}\ast 2\ast \left(\mathrm{s}.\mathrm{e}.\left(\widehat{\uptheta}\right)\right)/\mathrm{n},\kern0.5em \mathrm{G}\kern0.5em +\kern0.5em {\mathrm{t}}_{\upalpha /2}\ast 2\ast \left(\mathrm{s}.\mathrm{e}.\left(\widehat{\uptheta}\right)\right)/\mathrm{n}\right) $$

Where t_α/2_ = 1.645 for α = 0.05.
